# Validation of QSAR models for legislative purposes

**DOI:** 10.2478/v10102-009-0014-2

**Published:** 2009-09-28

**Authors:** Miloň Tichý, Marián Rucki

**Affiliations:** National Institute of Public Health, Šrobárova 48, 10042 Praha 10, Czech Republic

**Keywords:** alternative methods, toxicity testing, QSAR, validation

## Abstract

OECD principles of validation of Quantitative Structure – Activity Relationships (QSAR) models for legislative purposes are given and explained. Reasons of their origination and development, like system REACH, are described. A basic impulse has come from some OECD countries followed by all (almost) other countries of the world.

## Introduction

A long time it was clear that human and environmental health was necessary to protect against chemicals exposure. An important step was done in European Community (EC) when REACH (Registration, Evaluation, Authorisation and reduction of Chemicals) system has been originated and developed. The aim of REACH is to improve the protection of human and environmental health through the better and earlier identification of the intrinsic properties of chemical substances. REACH is European Community Regulation on chemicals and their safe use (EC 1907/2006). It deals with Registration, Evaluation, Authorisation and Reduction of Chemical substances. It entered into force of June 1, 2007, but discussed for years. The Regulation calls for the progressive substitution of the most dangerous chemicals when suitable alternatives have been identified. Manufacturers and importers are required to gather information on safety of chemicals using European Chemical Agency in Helsinki.

The former European Community legislative framework for chemical substances was a mix of many different national directives and regulations which have been developed historically. The hazard identification and risk assessment proved to be slow as well as the subsequent risk management. While new chemicals have to be tested before they are placed on the markets, there were no such provisions for existing chemicals. There was generally a lack of information publicly available in order to assess and control chemical substances effectively.

The two important aims are to improve protection of human health and environment from the risk of chemicals while enhancing the competitiveness of EC industry. This requires that industry has certain knowledge of the properties of its substances and manages potential risk.

## REACH and QSAR

The main principles of REACH are, thus, as follows. All chemicals are covered by REACH Regulation, unless they are explicitly exempted from its scope. The Regulation requires manufacturers and importers of chemicals to obtain relevant information on their substances and to use those data to manage them safely. A registration to the European Chemical Agency relates to each substance manufactures or imported in quantities of 1 tonne or above per year. Consequently toxicity testing with vertebrate animals has to be reduced or eliminated and data sharing is required for studies on such animals. The European Chemical Agency will manage the technical, scientific and administrative aspects of the REACH system, aiming to ensure that the legislation can be properly implemented and has credibility with all stakeholders.

Registration documents for performance requires to submit a technical dossiers for substances in quantities of 1 tonne and more and a chemical safety report for substances in quantities of 10 tonnes and more. The technical dossier contains information on the properties, uses and on the classification of a substance as well as guidance on safe use. To find the properties of substances, information requirements are set out in the testing annexes.

However, in practise a lack of data on both toxicological and physicochemical properties was perceptible fact. Data on more and more chemicals became necessity, but laboratory capacity to measure them was quite insufficient. New laboratories cannot be quickly built and equipped. Not speaking about laboratory animals and their farming.

New alternative methods of toxicity testing became desirable. The alternative method must make it possible to determine a toxic effect chosen in the same quality, if not better, as the traditional ones, to save laboratory animals, if not to eliminate their usage at all, to be quicker to satisfy demands of REACH, and, naturally, cheaper. General rules are also for set out for the use of existing information and techniques such as Quantitative Structure – Activity Relationships (QSAR) and read – across.

An establishment of an expert group at Organisation for Economic Co-operation and Development (OECD) was agreed during the 34. Joint Meeting of Chemical Committee and Working Party on Chemicals, Pesticides and Biotechnology in Paris, November 2002. Members of the expert group search for criteria and principles of legalisation for results of QSAR models for legislative purposes. Other meetings were organized simultaneously and consequently in Ispra (Italy) by European Centre for Validation of Alternative Methods at Joint Research Centre of European Committee (ECVAM/JRC(EC) in 2003, by joint organization by ECVAM and OECD in Paris 2004. The dealing on legalization of results obtained using QSAR models and techniques started in 2002 in Setubal (Portugal) as a consequence of other meetings on chemical safety to exposures and on principles for validation of *in vitro* methods (Setubal′s principles). A proposal of validation principles of (Q)SAR (meaning both SAR and QSAR methods) methods was a result of the meetings. The principles must be fulfilled for acceptation of the results of the QSAR methods for legislation purposes on the same level as results obtained with *in vitro* methods using low organisms, cells, organs, etc. The meetings of lawyers, state administrators, scientists of national, private and international organizations were always very vivid and interesting. The states were presented by their delegates in the whole spectrum of OECD: from Canada through EC to Japan and Korea. Besides QSAR methods, other alternative methods quicker than the traditional ones were looked for. The QSAR models were found as the best developed, known and scientifically based.

## OECD principles of models validation

A stormy discussion finally led to formulation of five “Setubal principles for QSAR validation”, after years taken discussions within the OECD committees nowadays “OECD principles (OECD, 2004).

1. **End-point measured must be transparently defined:**
*Inconvenience: the model can be constructed using data measured under different conditions and various experimental protocols.*
2. **The algorithm used for construction of a model must be univocally given.**
*Inconvenience: it is known that this information is not given with many commercial models. Information is not given, the organizations selling the model do not provide the information and it is not open to public. There are commercial reasons. This fact can be limited if some juristic decision should be made.*
3. **The applicability region must be defined.**
*Each QSAR model is directly joint with chemical structure of a molecule, with physicochemical properties of the substance and mechanism of the effect, which were used for a construction the model. All these parameters had values from some quantity to other one, depending mostly on availability of data and possibilities of their measurement. The same fact is valid for the biological test object, conditions of experiments, etc.*
4. **Suitable statistical evaluation of the models must exist.**
*Internal and external validation should be applied. The external validation with independent series of data should be used. If not possible cross-validation can also serve. The statistical indices joint with predictability and reproducibility of the model must be calculated.*
5. **A mechanism of the end-point effect should be given.**
*If known. This principle should push authors of the model to consider an interpretation of molecular descriptors used in construction of the model in mechanism of the effect and this study should be documented.*

The aim of these principles is to build models which could be able to be incorporated to legislative net in toxicity testing by manufacturers and distributors of chemical substances. This should satisfy even national authorities with respect for their specific demands.

## QSAR Tool Box and others

Softwares, expert systems and data system AMBIT were presented in the course organized in Ispra (Italy) in June 2007 by European Chemical Bureau, the organizations CEFIC (European Chemical Industry Council, European Committee) and OECD proposed independent software QSAR Tool Box working under Windows, required 2GHz processor or quicker, 8Gb on hard disc and 1 Gb for operating RAM. The development of Tool Box is paid by European Comunity and, thus, is available free of charge. This software should be friendly to administrators and less expensive.

A key part of Tool Box is so called categorization of chemicals. The categorization is ability of the system to group chemical substances to chemical categories. The chemical category is such a group of substances possessing similar physicochemical, toxicological and ecotoxicological properties or their fate in environmental and occupational surrounding or they behave using the common pattern as a result of chemical similarity.

## Statistical evaluation of predictivity of models

The QSAR models are formed mostly by linear regression analysis. Residual standard deviations (RSD) are a measure of deviations of estimated values by the model and experimental data. The quality of models can be evaluate by correlation coefficient squared (R^2^), coefficient of determination, or in the best, by external validation. Cross validation is used to judge the predictivity of the model, if no data remain for external validation. The original set of data is modified by taking off one (LOO – leave one out) or more (LMO – leave many out) data which are used to check a predictivity of a model formed from the remaining data. This process is repeated with all data in the set.

Correlation coefficient of regression between experimental and data estimated by cross validation is cross-validated correlation coefficient Q^2^. The QSAR model is “good” if Q^2^ > 0.5, excellent if Q^2^ > 0.9.

The predictivity of the model is evaluated by PRESS, which is a sum of differences between experimental and estimated data squared (Predictive REsidual Sum of Squares):

$$\text{PRESS}=\sum {\left(\exp-\text{cald}\right)}^{2}$$

Standard DEviation of Prediction (SDEP) is calculated from PRESS

$$\text{SDEP}={\left(\text{PRESS/n}\right)}^{½}$$

(Wold, 1991; Eriksson *et al*., 2003; Tichý, 2006; Tichý *et al*., 2005; OECD, 2006).

## References

[CIT0001] OECD, Environmental Directorate (2004). Report from the Expert group on quantitative structure – activity relationships (Q)SAR on the principles of validation of (Q)SARs.

[CIT0002] Wold S. (1991). Validation of QSARs. Quant Struct-Act Relat.

[CIT0003] Eriksson L, Jaworska J, Worth AP, Cronin MTD, McDowell RM (2003). Methods for reliability and uncertainty assessment and for applicability evaluations of classification- and regression-based QSARs. Environ Health Persp.

[CIT0004] Tichý M, Tichý M., Halouskková O. (2006). Validation of QSARs models [in Czech]. In Proceedings Alternative methods of toxicity testing of chemical substances and their mixtures (in Czech).

[CIT0005] Tichý M, Rucki M, Hanzlíková I, Čihák R, Dynterová A, Feltl L, Roth Z. (2005). Validation of alternative methods for hazard identification of chemical substances and their mixtures [in Czech]. České Pracov Lék.

[CIT0006] OECD Environmental Health and Safety Publication (2006). Series on Testing and Assessment. No. XX, Draft: Guidance document on the validation of (quantitative) structure – activity relationships models.

